# Tauroursodeoxycholic Acid Enhances the Quality of Postovulatory Aged Oocytes by Alleviating Oxidative Stress, Apoptosis, and Endoplasmic Reticulum Stress in Pigs

**DOI:** 10.3390/vetsci12030265

**Published:** 2025-03-12

**Authors:** Yan Wang, Jiayu Yuan, Chenran Sun, Ling Sun, Tao Lin

**Affiliations:** School of Life Sciences and Food Engineering, Hebei University of Engineering, Handan 056038, China; wangyan2022311@163.com (Y.W.); yjiayu03@163.com (J.Y.); schenran@126.com (C.S.); sunlingcnu@gmail.com (L.S.)

**Keywords:** tauroursodeoxycholic acid, postovulatory aged oocyte, ROS, apoptosis, endoplasmic reticulum stress

## Abstract

One of the major factors causing reduced developmental capacity of aged porcine oocytes is the induction of oxidative stress during oocyte aging. Tauroursodeoxycholic acid (TUDCA) supports cellular function by acting as an antioxidant and free radical scavenger. The aim of this study is to evaluate whether exogenous supplementation of TUDCA to the porcine in vitro maturation system can ameliorate the compromised quality of aged oocytes by mitigating free radical production. We found that TUDCA was able to effectively maintain normal oocyte morphology, cortical granule distribution, and spindle structure during postovulatory aging. Additionally, the blastocyst rate and total cell number in blastocysts were significantly increased in aged porcine oocytes treated with TUDCA. Importantly, aged porcine oocytes treated with TUDCA reduced ROS levels, increased the expression levels of GSH and *SOD1* genes, and improved the mitochondrial membrane potential ratio. Further study demonstrated that TUDCA significantly alleviated apoptosis in aged porcine oocytes, confirmed by the decreased Caspase 3 levels and ratio of *BAX* to *BCL2*. Interestingly, TUDCA could effectively alleviate the phenomenon of endoplasmic reticulum stress triggered during the oocyte aging process. Taking these findings together, our study demonstrates that TUDCA supplementation beneficially affects the quality of aged porcine oocytes by suppressing oxidative stress, apoptosis, and endoplasmic reticulum stress.

## Simple Summary

The objective of this study is to evaluate the effects of TUDCA on the quality of aged porcine oocytes. Our results show that the supplementation of TUDCA to the porcine in vitro maturation system can improve the quality of aged oocytes by maintaining normal oocyte morphology and mitochondrial membrane potential, as well as reducing ROS generation and apoptosis. Importantly, ER stress can be triggered during the oocyte aging process, and TUDCA supplementation can effectively alleviate this phenomenon. Taken together, our findings suggest that TUDCA could beneficially affect the quality of aged porcine oocytes by suppressing oxidative stress, apoptosis, and ER stress.

## 1. Introduction

One of the major factors causing reduced developmental capacity of aged porcine oocytes is the induction of oxidative stress during oocyte aging [1], as oxidative stress gives rise to a series of cascade reactions that negatively affect oocyte quality, such as morphological (spontaneous oocyte activation and oocyte death), cellular (spindle formation and cortical granule migration anomalies), and molecular (mitochondrial dysfunction and apoptosis) changes [2].

In addition to oocyte aging, standard oocyte and embryo operations in vitro (for example, in vitro oocyte maturation, in vitro fertilization, and somatic cell nuclear transfer) also lead to an accumulation of reactive oxygen species (ROS). Excessive ROS will trigger apoptosis, resulting in cell disintegration through the release of apoptogenic factors such as Caspase 3 [3]. Previous studies have shown that supplementation of exogenous antioxidants, such as Coenzyme Q10 [4], melatonin [5], bezafibrate [6], nicotinamide [7], and rapamycin [8], can effectively enhance the quality of aged oocytes and their development via their antioxidative and anti-apoptotic properties. Therefore, the balance between oxidative stress production and its detoxification is essential for maintaining oocyte quality during in vitro aging operations.

Tauroursodeoxycholic acid (TUDCA), an effective endoplasmic reticulum stress (ER stress) inhibitor [9,10], has been widely used to relieve endoplasmic reticulum stress during in vitro oocyte maturation and embryo development in a variety of species, such as pigs [11,12], mice [13], and bovines [14,15]. Moreover, TUDCA also serves another function in cells, acting as an antioxidant and free radical scavenger [10,16]. In mice, TUDCA treatment can alleviate tissue injury by reducing oxidative stress and apoptosis [17]. TUDCA supplementation in porcine cloning [12] or ICSI [18] embryos has been shown to reduce ROS levels and apoptosis while increasing the levels of the antioxidant Glutathione (GSH). Similarly, bovine embryos treated with TUDCA significantly reduced the levels of ROS and the pro-apoptotic *BAX* gene and increased the levels of the anti-apoptotic *BCL2* gene and GSH [19]. However, whether TUDCA can improve the quality and development of aged porcine oocytes by suppressing oxidative stress and apoptosis remains unclear.

Therefore, we designed an experiment to assess the effects of TUDCA supplementation on oxidative stress and apoptosis in porcine aged oocytes. In addition, considering that oxidative stress and the onset of ER stress are closely interconnected and often occur simultaneously [10,20], we hypothesized that ER stress might be induced during oocyte aging. Therefore, ER stress-related events were also investigated in aged porcine oocytes following TUDCA supplementation.

## 2. Materials and Methods

All chemicals and reagents employed in this study, unless stated otherwise, were obtained from Sigma-Aldrich (St. Louis, MO, USA).

### 2.1. Porcine Oocyte Collection and In Vitro Maturation (IVM)

Porcine ovaries were harvested and brought to the laboratory in 30–35 °C physiological saline supplemented with 1% (*v*/*v*) Penicillin–Streptomycin (2585609, Gibco, NY, USA) within 3 h of collection. Porcine follicular fluid was aspirated from 3 to 6 mm diameter follicles using a disposable syringe connected to an 18-gauge needle. Immature cumulus oocyte complexes (COCs) with uniform cytoplasm and at least three layers of cumulus cells were selected under a stereomicroscope for IVM. Approximately 50 COCs were cultured in each well of a 4-well dish with 500 μL maturation medium for 44 h at 38.5 °C, 5% CO_2_, and in air reaching saturated humidity. The maturation medium was based on Medium 199, consisting of 10% porcine follicular fluid (PFF), 1% Penicillin–Streptomycin (Gibco), 10 ng/mL epidermal growth factor (EGF), 10 IU/mL human chorionic gonadotropin (hCG), and 10 IU/mL equine chorion gonadotropins (eCGs). Oocytes displaying an integrated zona pellucida and homogeneous cytoplasm were considered normal, oocytes exhibiting cytoplasm cleavage after denuding were considered fragmented, and oocytes exhibiting lysis of the oolemma or damaged zona pellucida were considered dead. Only normal oocytes were used for the following study.

### 2.2. In Vitro Aging and Tauroursodeoxycholic Acid (TUDCA) Treatment

After 44 h IVM, the samples were transferred to a 0.1% hyaluronidase solution, and the cumulus cells surrounding the oocytes were removed using the vortexing method [5]. Oocytes with first polar bodies were collected and cultured in the same maturation medium under identical conditions for an additional 24 h to mimic postovulatory oocyte aging [5], and these oocytes were referred to as aged oocytes. To investigate the optimum concentration of TUDCA (T0266, Sigma, Darmstadt, Germany), oocytes were treated with TUDCA at different concentrations (0, 50, 200, and 500 μM) during the in vitro aging period.

### 2.3. Parthenogenetic Activation (PA) and In Vitro Culture (IVC) of Porcine Embryos

After 44 h IVM or in vitro aging, denuded oocytes were used for PA. For more details about the electrical situation, please refer to our previous description [21]. After PA, embryos were washed and cultured in PZM-3 medium with 5 μg/mL cytochalasin B (C6762, Sigma) for 4 h in an incubator to inhibit the secondary polar body extrusion. After 4 h of cytochalasin B treatment, embryos were transferred to PZM-3 medium supplemented with 3 mg/mL BSA for further culture. Cleavage embryos (>2-cell) and blastocysts were evaluated after 48 h and day 7, respectively. To count the total cell number, the blastocysts were collected and stained with DAPI.

### 2.4. Immunofluorescence Staining

The immunofluorescence staining of porcine oocytes was performed as previously described [22]. For evaluation of ROS and GSH, oocytes were treated with H2DCFDA (D399, Invitrogen, Carlsbad, CA, USA) and CMF2HC (C12881, Thermo Fisher Scientific, Waltham, MA, USA), respectively. Spindle and cortical granule (CG) distributions were stained using monoclonal anti-α-tubulin fluorescein isothiocyanate-labeled antibodies (F2168; Sigma) and FITC-PNA (L7381, Sigma-Aldrich). In a normal CG distribution, the CGs move to the cortex and establish a monolayer underneath the oolemma; however, in abnormal CG distributions, the CGs cannot form an integrated monolayer underneath the oolemma. In normal spindle formations, the spindle shows a shuttle shape and chromosome alignment at metaphase II plate; however, in abnormal spindle formations, the spindle cannot form a normal spindle structure or form a smaller size or has fewer microtubules. To identify mitochondrial membrane potential, the oocytes were stained with JC-1 dye (T3168; Thermo Fisher Scientific, Eugene, OR, USA). Apoptosis was evaluated using TUNEL assay (12156792910, In Situ Cell Death Detection Kit, TMR Red; Roche, Germany) and Caspase 3 antibody (sc-7272, Santa Cruz, California, USA). The chromosomes in the oocytes were stained with DAPI in VECTASHIELD Mounting Medium (H-1800, Vector Laboratories, Burlingame, CA, USA). For ROS and GSH analysis, samples were observed and photographed using an epifluorescence microscope (BX51, Olympus, Tokyo, Japan). In contrast, spindle, CGs, JC-1, TUNEL, and Caspase 3 analyses were conducted using a Zeiss laser scanning confocal microscope (Zeiss LSM 510, Oberkochen, Germany). ImageJ software (version 1.46r; National Institutes of Health, Bethesda, MD, USA) was used to evaluate the fluorescence intensities after background subtraction.

### 2.5. Real-Time PCR for Gene Expression Analysis

The details were described in a previously published study [23]. Briefly, total RNA was extracted from the oocytes using RNeasy Mini Kit (74104, Qiagen, Hilden, Germany), and complementary DNA was generated using TOPscript™ RT DryMIX kit (RT101, Enzynomics, Daejeon, South Korea). The real-time PCR reaction was performed using SYBR^®^ Premix Ex Taq™ kit (RR420A, Takara Bio Inc. Kusatsu, Japan) on a CFX96 Touch Real-Time PCR Detection System (Bio-Rad, California, USA). The 2^−ΔΔCt^ method was used to quantify the relative levels of the genes. The primers are listed in Table 1.

### 2.6. Statistical Analysis

Each independent experiment was performed with at least three biological replicates. The percentile data were processed with arcsine transformation, expressed as the mean ± standard error of the mean, and analyzed via one-way ANOVA followed by Fisher’s protected least significant difference test, using SPSS 26.0 software. *p* < 0.05 was considered statistically significant.

## 3. Results

### 3.1. TUDCA Improved Morphology and Development of Postovulatory Aged Oocytes

**Figure 1:**
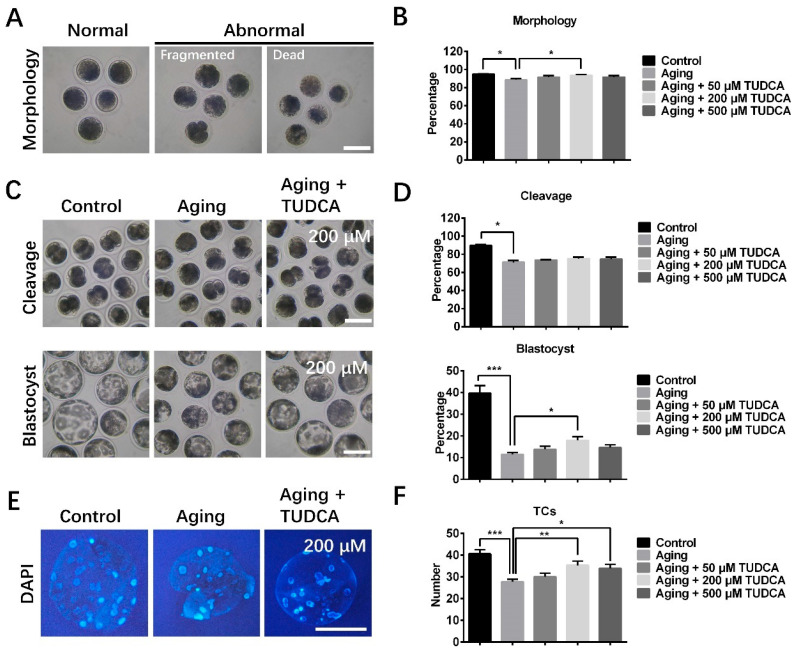
Influences of TUDCA on morphology and developmental potential of aged oocytes. (**A**) Typical images of normal, fragmented, and dead oocytes derived from the Control (i.e., fresh oocyte group), Aging (i.e., aged oocyte group), and Aging + TUDCA (i.e., TUDCA-treated aged oocyte group) groups. (**B**) The percentage of oocytes with normal morphology (Control n = 487; Aging n = 473; Aging + 50 μM TUDCA n = 484; Aging + 200 μM TUDCA n = 474; Aging + 500 μM TUDCA n = 463). (**C**) Representative images of cleavage- and blastocyst-stage embryos derived from control, aged, and TUDCA-treated oocyte groups. (**D**) The rate of cleavage and blastocyst formation (Control n = 164; Aging n = 156; Aging + 50 μM TUDCA n = 159; Aging + 200 μM TUDCA n = 162; Aging + 500 μM TUDCA n = 157). (**E**) Representative images of blastocysts after DAPI staining. (**F**) Numbers of total cells (TCs) in blastocysts. Bars = 100 μm. * *p* < 0.05, ** *p* < 0.01, *** *p* < 0.001.

As depicted in Figure 1, the percentage of oocytes with normal morphology (88.4 ± 1.6% vs. 94.9 ± 0.3%), cleavage (71.1 ± 2.0% vs. 89.6 ± 1.2%), and blastocyst formation (11.5 ± 0.9% vs. 39.7 ± 3.5%), and the total cell numbers in blastocyst (27.7 ± 1.2 vs. 40.6 ± 2.0) were remarkably lower in aged oocytes when compared with the control group (fresh group). The percentage of oocytes with normal morphology significantly increased in the group treated with 200 μM TUDCA compared to the aged control oocyte group and was maintained at levels similar to those in the fresh control group (93.4 ± 0.9% vs. 88.4 ± 1.6% and 94.9 ± 0.3%; Figure 1A,B). TUDCA did not affect the cleavage rates of aged oocytes, but the blastocyst rates in the 200 μM TUDCA group were significantly increased compared to the aged oocyte (Aging) group (17.9 ± 1.7% vs. 11.5 ± 0.9%; Figure 1C,D). Furthermore, both 200 μM and 500 μM TUDCA treatment groups exhibited an increase in total cell numbers within derived blastocysts when compared to the aged oocyte group (35.3 ± 2.0 and 33.9 ± 1.9 vs. 27.7 ± 1.2; Figure 1E,F). Based on these findings, subsequent experiments utilized a concentration of 200 μM TUDCA.

### 3.2. Effects of TUDCA on CG Distribution, Spindle Structure, and Nuclear Maturation in Aged Porcine Oocytes

**Figure 2:**
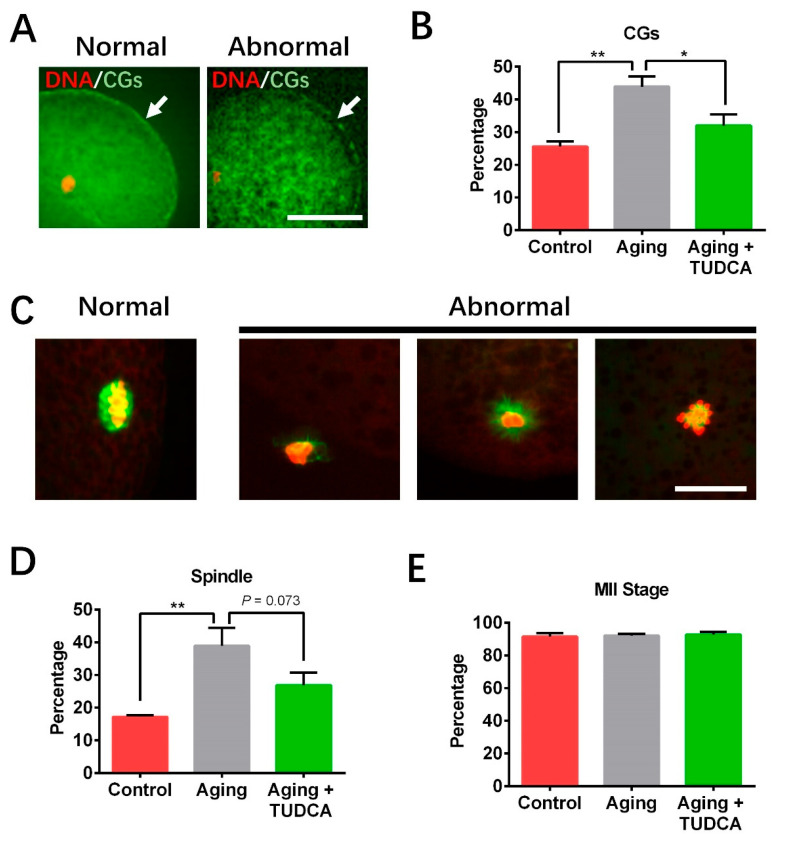
Influences of TUDCA on CG distribution, spindle formation, and nuclear maturation in aged porcine oocytes. (**A**) Typical pictures of CG distribution types. Normal CGs are shown on the left (arrow), and abnormal CGs are indicated on the right (arrow). (**B**) Percentage of abnormal CG distributions (Control n = 39; Aging n = 41; Aging + TUDCA n = 41). (**C**) Typical images of spindle structures. The red color represents DNA, and the green color indicates the spindle. (**D**) The percentages of abnormal spindle morphologies in oocytes (Control n = 36; Aging n = 36; Aging + TUDCA n = 33). (**E**) The proportions of oocytes that showed MII stage (Control n = 151; Aging n = 152; Aging + TUDCA n = 149). Scale bars represent 30 μm. * *p* < 0.05, ** *p* < 0.01.

The proportion of abnormal CG distributions was significantly higher in the aged oocyte groups than control groups (43.9 ± 3.1% vs. 25.5 ± 1.6%; Figure 2A,B). Meanwhile, TUDCA supplementation significantly decreased the rate of abnormal CG distribution when compared to aged oocytes not treated with TUDCA (32.0 ± 3.5% vs. 43.9 ± 3.1%; Figure 2A,B). The percentage of abnormal spindle morphology significantly increased in aged oocytes when compared with the control group (38.9 ± 5.6% vs. 17.2 ± 0.5%; Figure 2C,D). Although TUDCA supplementation could decrease the rate of abnormal spindle morphologies in aged oocytes, the difference was not significant (26.9 ± 3.9% vs. 38.9 ± 5.6%, *p* = 0.073; Figure 2C,D). In addition, there was no difference in the nuclear maturation (MII stage) rate among the control, aged, and TUDCA oocyte groups (91.5 ± 2.3% vs. 92.1 ± 1.1% vs. 92.7 ± 1.6%; Figure 2E).

### 3.3. TUDCA Promoted Antioxidative Ability in Aged Porcine Oocytes

**Figure 3:**
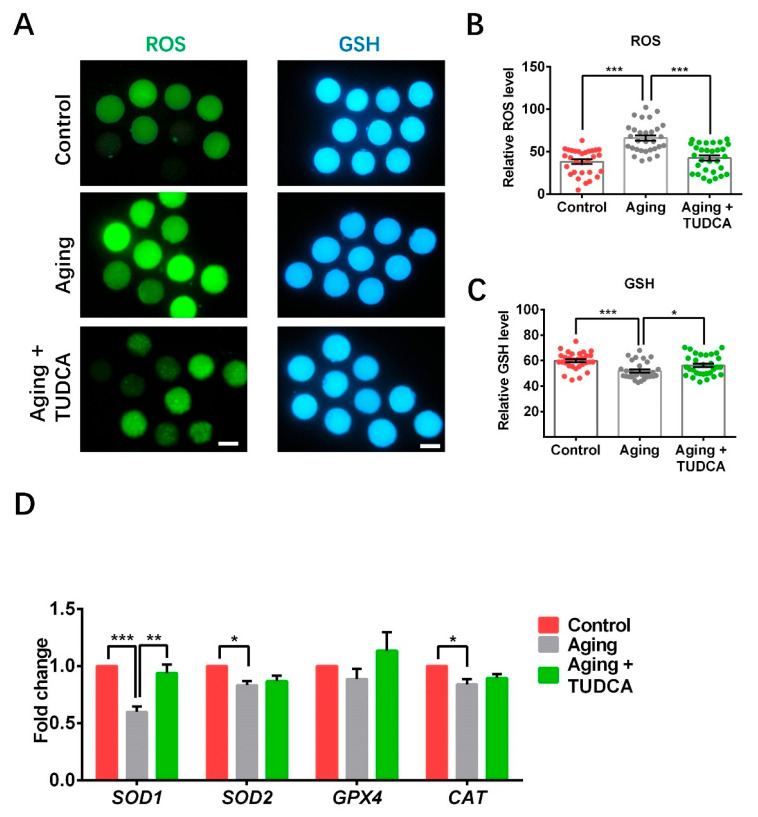
TUDCA enhanced the antioxidative ability in aged porcine oocytes. (**A**) Typical images of oocytes stained with H2DCFDA (green color) and CellTracker Blue CMF2HC (blue color). (**B**) Fluorescence intensity of ROS staining (Control n = 30; Aging n = 30; Aging + TUDCA n = 30). (**C**) Fluorescence intensity of GSH staining (Control n = 30; Aging n = 30; Aging + TUDCA n = 30). (**D**) Expression levels of antioxidative ability-related genes. Scale bars = 100 μm in (**A**). * *p* < 0.05. ** *p* < 0.01, *** *p* < 0.001.

The levels of ROS were significantly increased in aged oocytes compared to the control (66.0 ± 3.2 vs. 38.3 ± 2.7; Figure 3A,B). However, aged oocytes treated with TUDCA exhibited significantly lower ROS levels compared to the untreated aged oocyte group (42.6 ± 3.0 vs. 66.0 ± 3.2; Figure 3A,B). In contrast, the levels of GSH were significantly reduced in aged oocytes compared to the control (51.9 ± 1.2 vs. 59.8 ± 1.3; Figure 3A,C). TUDCA treatment, on the other hand, led to significantly higher GSH levels compared to the untreated aged oocyte group (56.2 ± 1.5 vs. 51.9 ± 1.2; Figure 3A,C). The levels of *SOD1*, *SOD2*, and *CAT* were remarkably reduced in the aged oocytes group when compared with the control (0.60 ± 0.05 vs. 1.00 ± 0.00; 0.83 ± 0.04 vs. 1.00 ± 0.00; 0.84 ± 0.05 vs. 1.00 ± 0.00; Figure 3D). TUDCA treatment did not influence *SOD2* and *CAT* expression levels, while it significantly increased the expression level of *SOD1* compared to untreated aged oocytes (0.94 ± 0.07 vs. 0.60 ± 0.05; Figure 3D). Additionally, there was no difference in the expression levels of *GPX4* among these groups (Figure 3D).

### 3.4. TUDCA Prevented the Reduction in Mitochondrial Membrane Potential in Aged Porcine Oocytes

**Figure 4:**
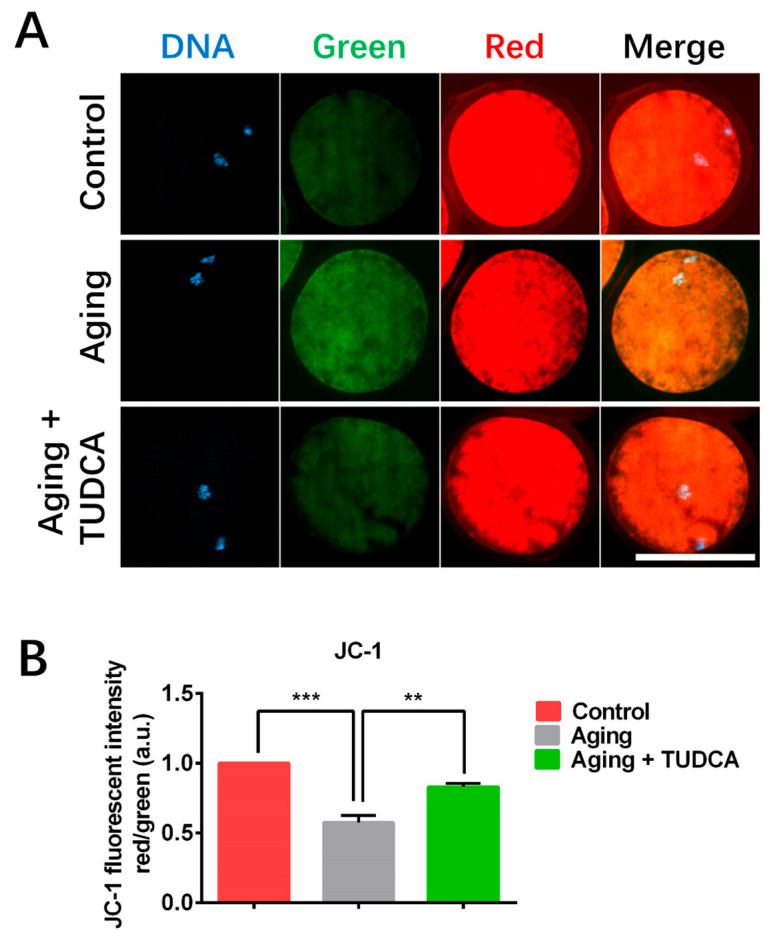
Influences of TUDCA on mitochondrial membrane potential in aged porcine oocytes. (**A**) JC-1 staining. (**B**) JC-1 fluorescence intensity (red/green ratio) (Control n = 45; Aging n = 45; Aging + TUDCA n = 45). Scale bar = 100 μm. ** *p* < 0.01, *** *p* < 0.001.

The mitochondrial membrane potential was evaluated in oocytes using the JC-1 fluorescence probe. The JC-1 fluorescence intensity (red/green ratio) was significantly lower in aged oocytes when compared to the fresh control oocytes (0.57 ± 0.05 vs. 1.00 ± 0.00; Figure 4). However, TUDCA significantly increased mitochondrial membrane potential (red/green ratio) in aged porcine oocytes compared to aged oocytes (0.83 ± 0.02 vs. 0.57 ± 0.05; Figure 4).

### 3.5. TUDCA Decreased Apoptosis in Aged Porcine Oocytes

**Figure 5:**
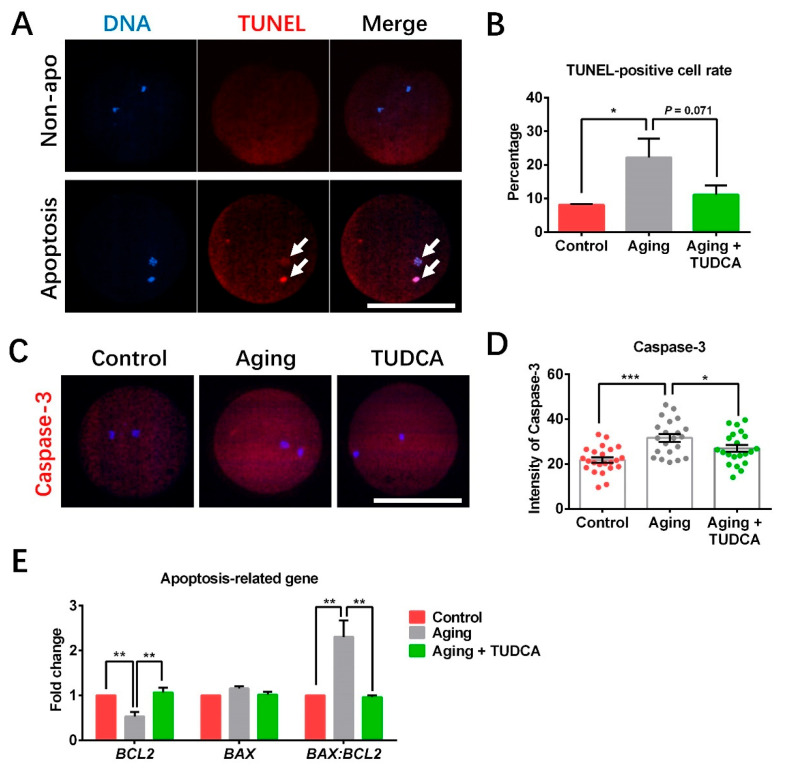
TUDCA decreased apoptosis levels in aged porcine oocytes. (**A**) Typical images of oocytes stained with TUNEL, apoptotic cells stained in red (white arrows), and DNA stained in blue. (**B**) TUNEL-positive cell rate (Control n = 37; Aging n = 36; Aging + TUDCA n = 36). (**C**) Images of porcine oocytes stained with Caspase 3. (**D**) Fluorescence intensity of Caspase 3 (Control n = 23; Aging n = 21; Aging + TUDCA n = 21). (**E**) The expression levels of apoptosis-related genes. Scale bars = 100 μm. * *p* < 0.05. ** *p* < 0.01, *** *p* < 0.001.

The TUNEL assay showed that the TUNEL-positive cell rate was dramatically increased in the aged oocyte group compared to that in the control group (22.2 ± 5.6% vs. 8.1 ± 2.1%) but decreased (*p* = 0.071) in the TUDCA-treated aged oocyte group (11.1 ± 2.8%; Figure 5A,B). Aged oocytes showed significantly increased Caspase 3 expression levels when compared to fresh (control) oocytes (31.7 ± 1.7 vs. 21.8 ± 1.2); however, TUDCA supplementation was able to significantly decrease the Caspase 3 level in aged oocytes (27.0 ± 1.6, *p* < 0.05; Figure 5C,D). The level of anti-apoptosis gene *BCL2* dramatically decreased in the aged oocyte compared to that in the control (0.53 ± 0.10 vs. 1.00 ± 0.00) but increased in the TUDCA-treated aged oocyte group (1.07 ± 0.11; Figure 5E). TUDCA significantly decreased the ratio of *BAX* to *BCL2* compared with the aged oocyte group (0.96 ± 0.04 vs. 2.30 ± 0.37; Figure 5E). Except for Caspase 3 expression levels (27.0 ± 1.6 vs. 21.8 ± 1.2, *p*< 0.05), no differences were observed in the TUNEL-positive cell rate (11.1 ± 2.8% vs. 8.1 ± 2.1%), the expression levels of *BCL2* (1.07 ± 0.11 vs. 1.00 ± 0.00) and *BAX* (1.02 ± 0.06 vs. 1.00 ± 0.00), and the ratio of *BAX* to *BCL2* (0.96 ± 0.04 vs. 1.00 ± 0.00) between the TUDCA-treated aged oocyte group and the control group.

### 3.6. TUDCA Enhances the Quality of Postovulatory Aged Oocytes by Alleviating ER Stress

**Figure 6:**
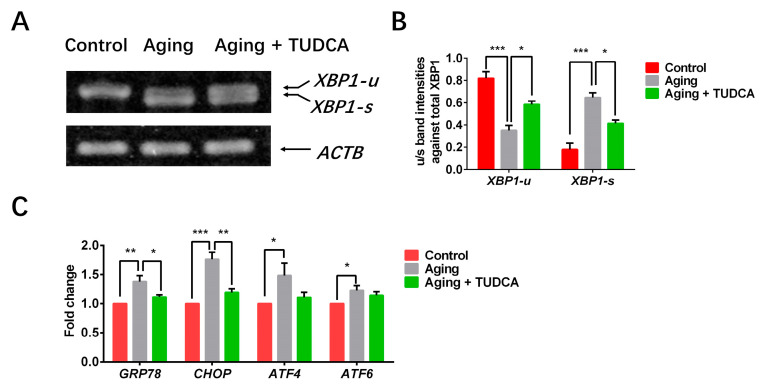
TUDCA improved the quality of aged oocytes by alleviating ER stress. (**A**,**B**) Expression patterns (**A**) and levels (**B**) of *XBP1-u* and *XBP1-s* genes in aged oocytes treated with TUDCA. (**C**) Expression levels of ER stress-related genes. * *p* < 0.05. ** *p* < 0.01, *** *p* < 0.001 (Figure S1).

To verify our hypothesis that ER stress can be triggered during oocyte aging, we investigated the expression of ER stress-related genes in aged porcine oocytes following TUDCA treatment. As shown in Figure 6, the expression levels of *XBP1-s* against total *XBP1* mRNA, *GRP78*, *CHOP*, *ATF4*, and *ATF6* were significantly higher in the aged oocyte group compared to the controls (0.65 ± 0.04 vs. 0.18 ± 0.06; 1.38 ± 0.10 vs. 1.00 ± 0.00; 1.76 ± 0.12 vs. 1.00 ± 0.00; 1.48 ± 0.21 vs. 1.00 ± 0.00; 1.23 ± 0.08 vs. 1.00 ± 0.00). However, TUDCA supplementation significantly reduced the expression levels of *XBP1-s* against total *XBP1* mRNA (0.41 ± 0.03), as well as *GRP78* (1.11 ± 0.04) and *CHOP* (1.19 ± 0.06), which were induced by oocyte aging. Except for the expression levels of *XBP1-s* against total *XBP1* mRNA (0.41 ± 0.03 vs. 0.18 ± 0.06, *p* < 0.05), no differences were observed in the expression levels of *GRP78* (1.11 ± 0.04 vs. 1.00 ± 0.00), *CHOP* (1.19 ± 0.06 vs. 1.00 ± 0.00), *ATF4* (1.11 ± 0.09 vs. 1.00 ± 0.00) and *ATF6* (1.14 ± 0.06 vs. 1.00 ± 0.00) genes between the TUDCA-treated aged oocyte group and the control group.

### 3.7. TUDCA Decreased ROS Level and Apoptosis, Increased GSH Level in Porcine Oocytes Induced by H_2_O_2_

**Figure 7:**
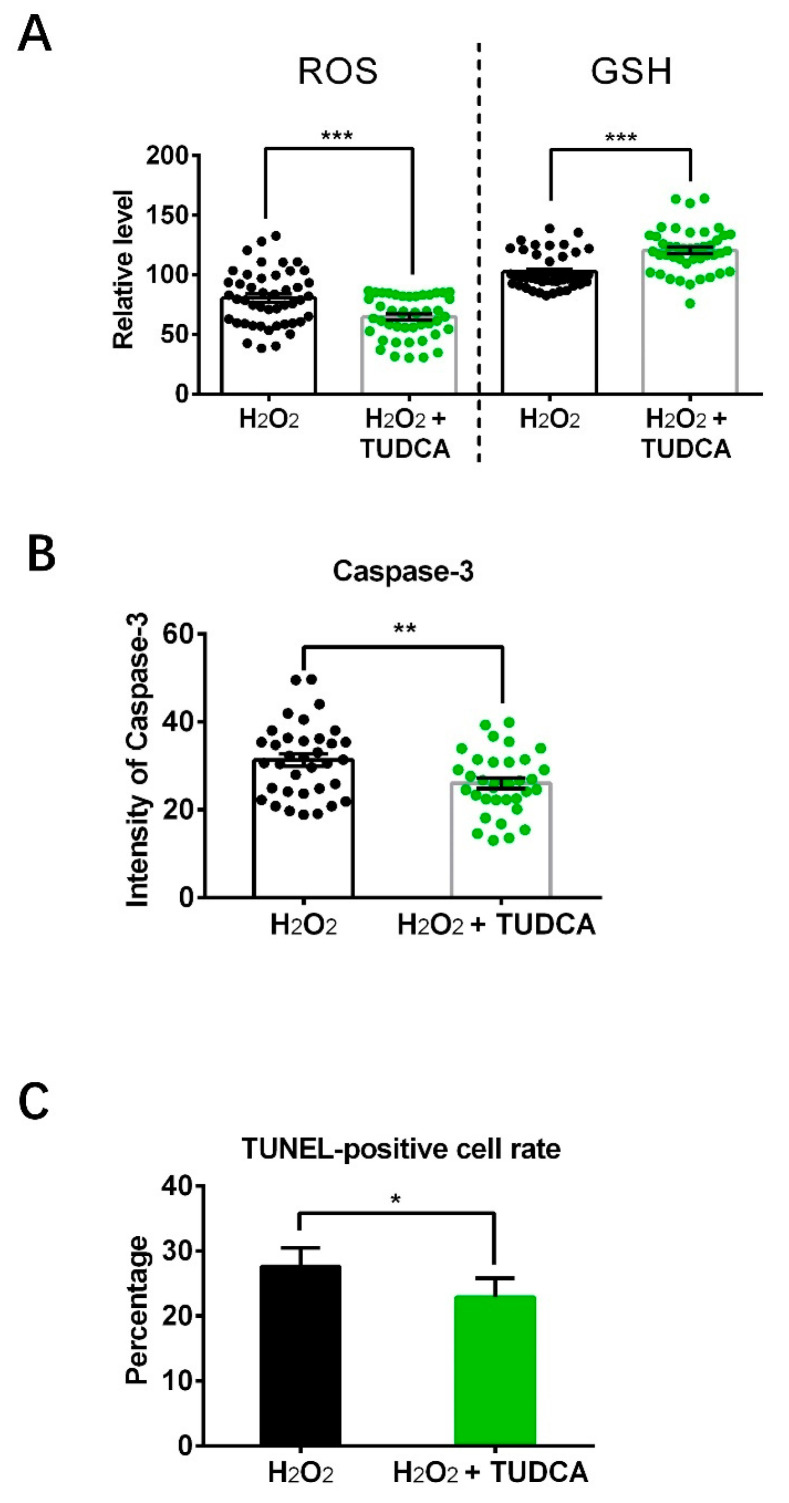
The levels of ROS, GSH, Caspase 3, and TUNEL-positive cell rate in oocytes following H_2_O_2_ and TUDCA treatment. (**A**) Fluorescence intensity of ROS (H_2_O_2_ n = 46; H_2_O_2_ + TUDCA n = 45) and GSH (H_2_O_2_ n = 45; H_2_O_2_ + TUDCA n = 45) staining. (**B**) Fluorescence intensity of Caspase 3 (H_2_O_2_ n = 35; H_2_O_2_ + TUDCA n = 35). (**C**) TUNEL-positive cell rate (H_2_O_2_ n = 43; H_2_O_2_ + TUDCA n = 44). * *p* < 0.05, ** *p* < 0.01, *** *p* < 0.001.

Finally, to clarify that the rescue effects of TUDCA on aged porcine oocytes were indeed working through the elimination of oxidative stress, the levels of ROS and GSH, and apoptosis were assessed in the oocytes following treatment with H_2_O_2_. As shown in Figure 7, compared to the H_2_O_2_ treatment groups, oocytes co-treated with TUDCA showed significantly decreased levels of ROS (64.6 ± 2.6 vs. 80.5 ± 3.4) and Caspase 3 (26.0 ± 1.2 vs. 31.3 ± 1.4), decreased TUNEL-positive signaling rate (22.9 ± 2.9% vs. 27.7 ± 2.8%), and increased GSH level (120.5 ± 2.7 vs. 102.6 ± 2.2).

## 4. Discussion

In this study, we investigated the effects of TUDCA supplementation on the quality of postovulatory aged oocytes in pigs. We found that TUDCA could act as a powerful inhibitor to delay oocyte aging by maintaining normal oocyte morphology, CG distribution, spindle structure, and mitochondrial membrane potential, as well as reducing ROS, apoptosis levels, and ER stress.

The morphology of oocytes has been reported as a key indicator of oocyte quality [2,24,25]. Numerous studies have reported that cytoplasmic fragmentation occurs during oocyte aging, resulting in a loss of developmental potential [2,5,24]. Consistent with previous studies, we found a high proportion of abnormal morphological phenotypes, including fragmentation and cell death, in aged oocytes compared to the fresh oocyte group. However, TUDCA (200 μM) supplementation improved the normal oocyte rates in aged oocytes, resulting in morphological phenotypes similar to those observed in fresh oocytes. Further study showed that the rate of blastocyst formation and the total number of cells within the blastocysts were improved in aged oocytes treated with TUDCA, suggesting that TUDCA not only has the potential to enhance the quality of aged oocytes, but also can rescue the poor developmental capacity of aged porcine oocytes.

To further investigate how TUDCA reverses aging-related decline in oocyte quality and developmental potential, we examined key indicators of oocyte quality, including spindle structure and CG distribution in aged porcine oocytes. The spindle is a crucial element of the cytoskeleton that plays a critical role in chromosome distribution [26]. Cortical granules (CGs), which are oocyte-specific vesicles, function to prevent polyspermy [7]. Proper CG dynamics are often considered an indicator of oocyte cytoplasmic maturation. It has been reported that oocyte aging damages spindle assembly and CG distribution [7,24,27], resulting in reduced oocyte quality. Here, we confirm these phenomena and suggest that TUDCA supplementation could prevent abnormal spindle formation and CG distribution during oocyte aging.

Mitochondria are critical for maintaining cellular metabolic function [28], and abnormal mitochondrial integrity and function can be altered during the oocyte aging process, triggering oxidative stress and apoptosis and further negatively influencing subsequent fertilization and development [29,30]. Our findings revealed that mitochondrial membrane potential significantly decreases in aged oocytes when compared to the fresh control group, and TUDCA supplementation could ensure mitochondrial membrane potential remained in a normal state in aged porcine oocytes.

One major factor contributing to the impaired development of aged oocytes is the generation of oxidative stress during postovulatory aging [31], which triggers various detrimental cascades that compromise oocyte quality. Previous studies have demonstrated that TUDCA can reduce oxidative stress by decreasing ROS levels and enhancing GSH levels in various species, including mice [32], pig [12,18,33], and cattle [34,35]. Here, we found that the levels of ROS in aged oocytes were significantly higher due to the prolonged in vitro maturation time; however, TUDCA supplementation could decrease ROS levels and increase GSH expression relative to aged control oocytes. Superoxide radicals are initially converted into hydrogen peroxide by SOD, which is then further detoxified into harmless molecules, such as water and oxygen, through the actions of CAT and GPX [36]. This process is crucial for preserving normal cellular redox balance, preventing the formation of free radicals. Oocyte aging has been reported in previous studies to decrease the expression levels of antioxidative stress genes (*SOD*, *GPX4*, and *CAT*, etc.) [5,37]. Consistent with previous studies, *SOD1*, *SOD2*, and *CAT* genes were significantly downregulated in aged oocytes; however, TUDCA could increase the expression of *SOD1* in aged porcine oocytes. These results suggest that TUDCA supplementation may suppress oxidative stress in aged porcine oocytes by reducing ROS production and enhancing the levels of GSH and *SOD1* gene expression.

Previous studies have reported that excessive ROS induces apoptosis via mitochondrial apoptogenic factors such as Caspase 3 [6,38]. We examined the influence of TUDCA on apoptosis in aged porcine oocytes by TUNEL assay and immunofluorescence methods. We found that aged oocytes treated with TUDCA not only reduced TUNEL-positive signaling rates but also reduced Caspase 3 expression. Members of the BCL-2 gene family play a vital role in regulating cell apoptosis by controlling cytochrome c release and apoptosome assembly [39]. Within the BCL-2 family, the pro-apoptotic *BAX* gene and the anti-apoptotic *BCL-2* genes regulate apoptotic pathways during embryo development [40]. To further clarify the underlying molecular mechanisms of TUDCA reducing apoptosis in aged porcine oocytes, we next examined the expression of *BCL2* and *BAX* using real-time PCR. The results indicated that TUDCA increased the levels of *BCL2* and decreased the *BAX* to *BCL2* ratio in aged oocytes, which suggested that TUDCA prevents apoptosis in aged porcine oocytes.

Exposure to H_2_O_2_ leads to the accumulation of intracellular ROS, inducing oxidative stress [41]. To confirm that the rescue effects of TUDCA on aged porcine oocytes are achieved specifically through the reduction in oxidative stress, we investigated the levels of ROS and apoptosis in H_2_O_2_-supplemented oocytes following TUDCA treatment. TUDCA not only decreased H_2_O_2_-induced ROS levels and increased GSH levels, but also suppressed apoptosis by reducing the TUNEL-positive cell rate and Caspase 3 expression, suggesting that TUDCA acts as an antioxidant.

ROS gradually accumulates in oocytes with extended aging time following ovulation both in vitro and in vivo [42]. Oxidative stress can trigger ER stress [20]. Therefore, we speculated that ER stress might be induced during oocyte aging due to the accumulation of ROS generated in the aging oocytes. Here, we confirmed the presence of ER stress during in vitro oocyte aging; however, TUDCA was found to effectively alleviate this stress.

X-box binding protein 1 (XBP1) is an important regulator of a specific subset of genes activated during ER stress [32]. Under stress conditions, XBP1 transcription is activated, leading to the production of spliced XBP1 (*XBP1-s*) from its unspliced form (*XBP1-u*). Importantly, only *XBP1-s* can translocate into the nucleus to regulate genes involved in the unfolded protein response (UPR). Therefore, *XBP1-s* is widely used as a marker to monitor ER stress [43]. We found that the spliced form of XBP1 product was clearly detected in aged porcine oocytes compared to the fresh oocyte control group, suggesting that ER stress was triggered during oocyte aging in vitro. This was confirmed by our experimental data, which showed that the levels of ER stress-related genes, particularly *CHOP*, *GRP78*, *ATF4* and *ATF6*, were upregulated in aged porcine oocytes. The supplementation of TUDCA during the oocyte aging period reduced the expression levels of ER stress-related genes (especially *XBP1-s*, *CHOP*, and *GRP78*), suggesting that TUDCA could restore porcine oocyte quality by reversing aging-induced ER stress.

## 5. Conclusions

In summary, our study demonstrated that TUDCA supplementation effectively improved the quality and development of aged porcine oocytes by maintaining oocyte morphology and alleviating oxidative stress, apoptosis, and ER stress. These results provide valuable insights that could potentially be used to delay oocyte aging in other animal species or in humans.

## Figures and Tables

**Table 1:** Primer sequences for PCR.

Gene	Primer Sequence 5′-3′	Length (bp)	Access No./Reference
*SOD1*	F: TCCATGTCCATCAGTTTGGA R: AGTCACATTGGCCCAGGTCTC	131	[5]
*GPX4*	F: ATTCTCAGCCAAGGACATCG R: CCTCATTGAGAGGCCACATT	93	[5]
*CAT*	F: ACATGGTCTGGGATTCTGG R: TCATGTGCCTGTGCCATCT	99	[5]
*SOD2*	F: GGTGGAGGCCACATCAATCA R: AACAAGCGGCAATCTGCAAG	220	NM_214127.2
*BAX*	F: ACACCTCATAGCCATGAAAC R: ATGGCTGACATCAAGATACC	232	YA_55048
*BCL2*	F: AGAGCTTTGAGCAGGTATTG R: GCATTGTTTCCGTAGAGTTC	253	NM_214285
*XBP1*	F: AACGATCAAGCAGTGACTATTCG R: GAGTACAGGGTGGTGAAGTGAGG	263	AF074419
*GRP78*	F: CGGAGGAGGAGGACAAGAAGGAG R: ATATGACGGCGTGATGCGGTTG	143	XM_001927795.7
*CHOP*	F: AAGACCCAGGAAACGGAAAC R: TCCAGGAAAGGTCAGCAGTA	261	NM_001144845.1
*ATF4*	F: TGAGCCCTGACTCCTATCTG R: TCCAGCTCTTTACATTCGCC	277	NM_001123078.1
*ATF6*	F: GGAGTTAAGACAGCGCTTGG R: GAGATGTTCTGGAGGGGTGA	142	NM_001271738.1
*ACTB*	F: GTGGACATCAGGAAGGACCTCTA R: ATGATCTTGATCTTCATGGTGCT	137	U_07786

F, Forward primer; R, Reverse primer.

## Data Availability

All the data are presented in the manuscript.

## Supplementary Materials

The following supporting information can be downloaded at: https://www.mdpi.com/article/10.3390/vetsci12030265/s1, Figure S1: the original image for Xbp1 gene.
