# Supplementary figures and images for: Tauroursodeoxycholic Acid Enhances the Quality of Postovulatory Aged Oocytes by Alleviating Oxidative Stress, Apoptosis, and Endoplasmic Reticulum Stress in Pigs
Source: Vet Sci. 2025 Mar 12;12(3):265. doi: 10.3390/vetsci12030265 (PMC11946076; doi:10.3390/vetsci12030265)

Figure S1. The original images for Xbp1 gene.

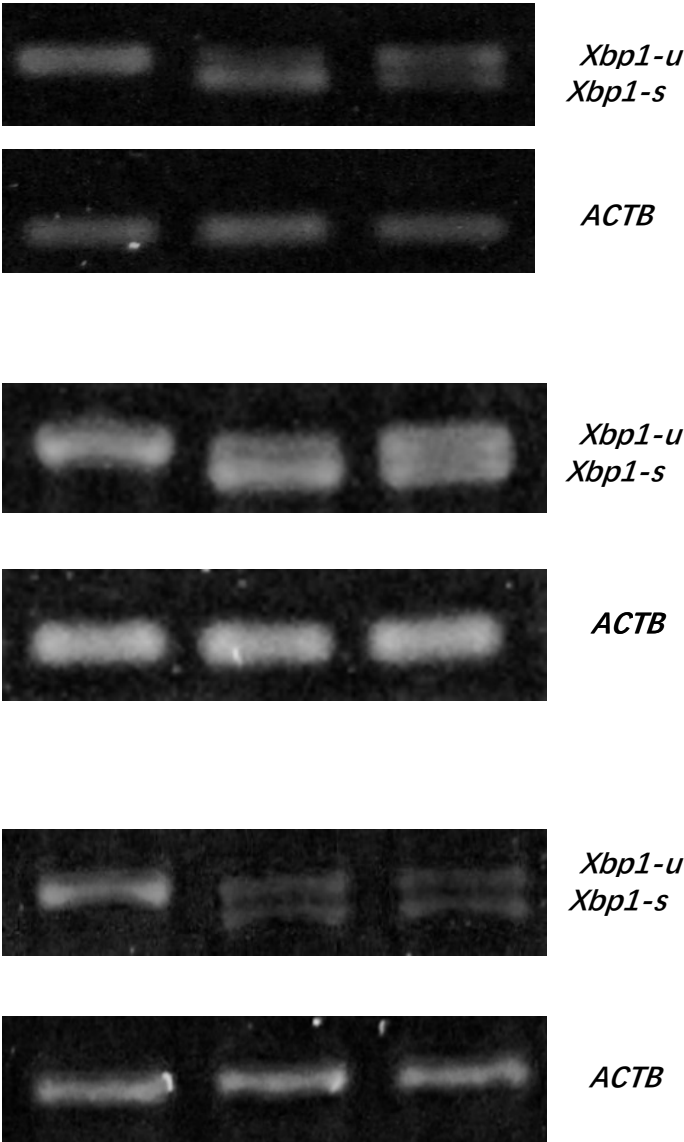

Supplement: Supplementary file 1 [file vetsci-12-00265-s001.zip › vetsci-3469173-supplementary.pdf]
